# Assessing and Modelling the Efficacy of *Lemna paucicostata* for the Phytoremediation of Petroleum Hydrocarbons in Crude Oil-Contaminated Wetlands

**DOI:** 10.1038/s41598-020-65389-z

**Published:** 2020-05-22

**Authors:** Abraham Ogheneruemu Ekperusi, Eunice Oluchi Nwachukwu, Francis David Sikoki

**Affiliations:** 1 Africa Centre of Excellence, Centre for Oilfield Chemicals Research, Institute of Petroleum Studies, University of Port Harcourt, Choba, Rivers State Nigeria; 2 Department of Marine Environment & Pollution Control, Faculty of Marine Environmental Management, Nigeria Maritime University, Okerenkoko, Delta State Nigeria; 3 Department of Plant Science & Biotechnology, Faculty of Science, University of Port Harcourt, Choba, Rivers State Nigeria; 4 Department of Animal & Environmental Biology, Faculty of Science, University of Port Harcourt, Choba, Rivers State Nigeria

**Keywords:** Environmental biotechnology, Biogeochemistry, Environmental sciences

## Abstract

The potentials of the invasive duckweed species, *Lemna paucicostata* to remove pollutants from aquatic environment was tested in a constructed wetlands as an ecological based system for the phytoremediation of petroleum hydrocarbons in crude oil-contaminated waters within 120 days. Total petroleum hydrocarbons in wetlands and tissues of duckweed were analyzed using gas chromatography with flame ionization detector following established methods while the experimental data were subjected to the first-order kinetic rate model to understand the remediation rate of duckweed in wetlands. *L. paucicostata* effected a significant (F = 253.405, P < 0.05) removal of hydrocarbons from wetlands reaching 97.91% after 120 days. Assessment on the transport and fate of hydrocarbons in duckweed indicated that *L. paucicostata* bioaccumulated less than 1% and significantly biodegraded 97.74% of hydrocarbons in wetlands at the end of the study. The experimental data reasonably fitted (r^2^ = 0.938) into the first-order kinetic rate model. From the result of the study, it is reasonable to infer that *L. paucicostata* is an effective aquatic macrophyte for the removal of petroleum hydrocarbons in moderately polluted waters.

## Introduction

The expansion of the chemical industry after the dawn of the industrial age has significantly increased the levels of contaminants entering the natural and human environment. Among the many chemical industries, the petrochemical industry is a major player in the global energy landscape despite significant investment and interest in alternative energy. Today, petroleum remains a significant energy demand in modern society. In spite of all the necessary safety and precautionary approach in the petroleum industry, oil spill is an inevitable occurrence in the production, transport, processing and consumption of crude oil^1,2^. The continuous advancement in offshore exploration has increased the level of exposure of hydrocarbons into aquatic environment, particularly freshwater, creating a cascade of negative impact on organisms relying on freshwater resources^3,4^. Oil spills in water leads to extensive damage of water resources including sensitive habitats. It suffocates aquatic life and renders water unfit for communal and domestic use^5^. Over the years, various technological approach has been developed for the treatment of oil spill in marine and coastal waters, as well as rivers, streams, wetlands, swamps and lakes^6–9^. Chemical methods involving the application of dispersants and *in situ* burning have unintended consequences despite the success recorded with such application^8,10^. The unintended effects with the application of chemicals for oil spill cleanup have given opportunities to the development of biological and nature-based solutions over the past three decades for the remediation of contaminants in aquatic environment^7,11^.

The usefulness of plants in the biological remediation of contaminants has been expanding over the years. Several species of plants with the potentials to remove a wide range of contaminants from the environment have been documented in the literature^6,11,12^. For water pollution, various macrophytes have been investigated for the uptake, bioaccumulation and degradation of a wide range of chemical pollutants in natural and man-made wetlands^9,11,13,14^. Macrophytes such as duckweeds, water lettuce and water hyacinth found exclusively on the surface of water bodies have been reported to remediate nutrients load, heavy metals and hydrocarbons in wetlands^11^.

Among the various surface floating macrophytes groups, duckweeds species have been used extensively for the phytoremediation of pollutants. Their invasive nature, wide distribution, simple structure and sporadic growth pattern and the ability to thrive in diverse habitat and tolerate a high level of contaminants in the environment are some of the unique features that make duckweeds suitable candidates for pollutants uptake and removal in surface waters^11,15,16^. Duckweeds have a long history of application in aquaculture, livestock production, poultry, pharmaceuticals and cosmetics, medicine, biofuels, toxicity testing, environmental monitoring and for the remediation of organic compounds in contaminated wetlands^11,17,18^. Prominent members of the group applied for remediation research are found in the *Lemna* genera. *Lemna* species have been successfully applied in wetlands for the removal of a wide range of pollutants, particularly organic nutrients and heavy metals^19–21^. Previous workers have indicated the removal of organic compounds like phenol^22^, chlorobenzotriazole^9^, benzotriazoles^23^ and anthracene, phenanthrene and benzo[a]pyrene^24^ by various *Lemna* species but there is paucity of literature on the removal of petroleum hydrocarbons by *L. paucicostata* (Hegelm.). This study aimed to assess and model the efficacy of duckweed, *L. paucicostata* for the phytoremediation of crude oil-contaminated waters and to determine the transport and fate of the pollutants in *L. paucicostata*.

## Materials and Method

### Plant culture and identification

**Figure 1:**
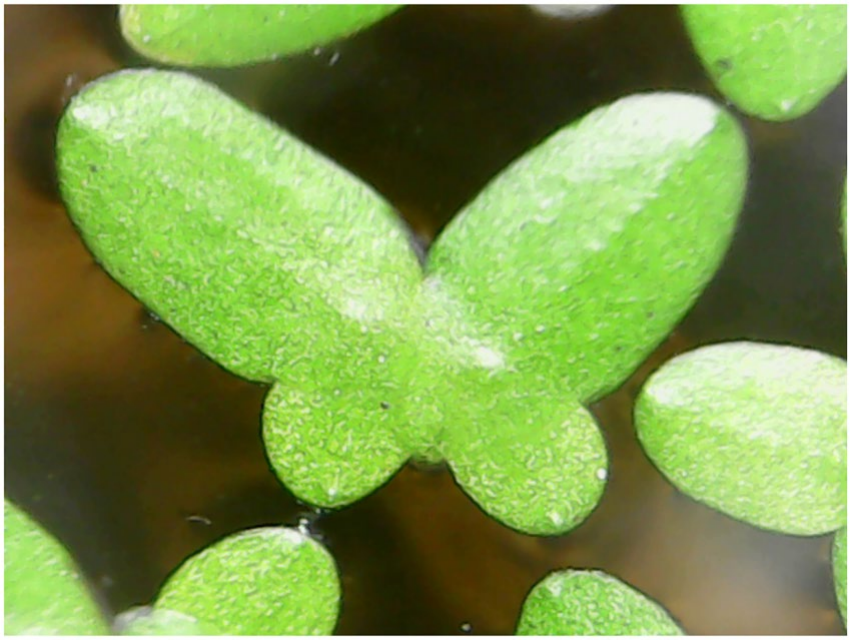
Adult duckweed with emerging daughter fronds.

Colonies of *L. paucicostata* were collected from drains at Effurun, Delta State, Nigeria and were cultured using Zyme nutrient solution. Duckweed was acclimated for seven days before introduction into experimental setup (Fig. 1). Identification of duckweed was carried out using historical references aided with appropriate taxonomic keys^25–27^. Environmental conditions such as temperature, photoperiod and relative humidity were monitored all through the culture and experimental period.

### Experimental setup

**Figure 2:**
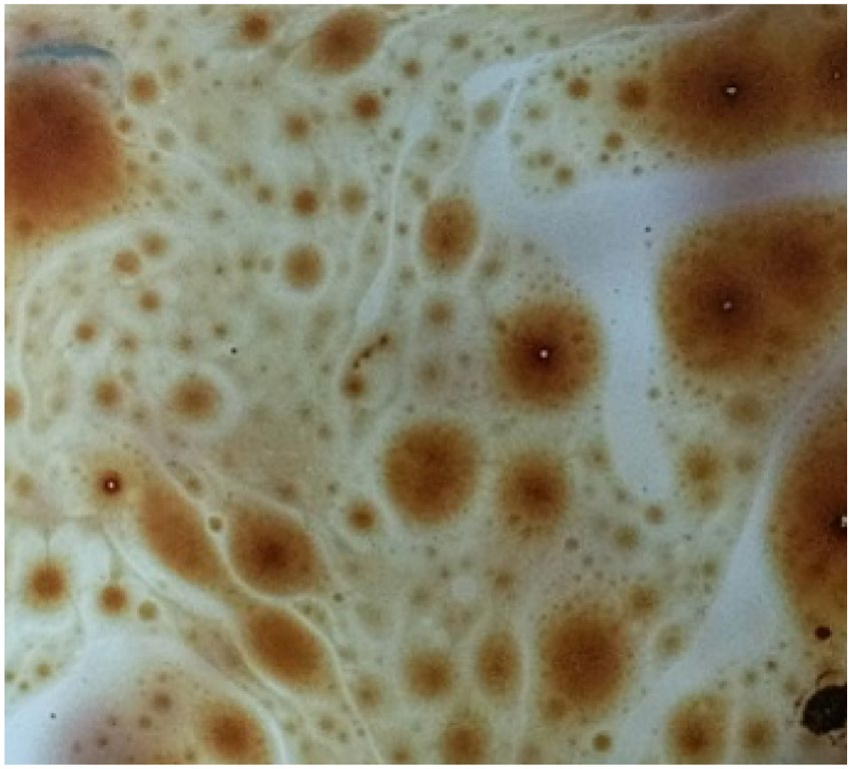
Floating crude oil in surface of the contaminated wetlands.

**Figure 3:**
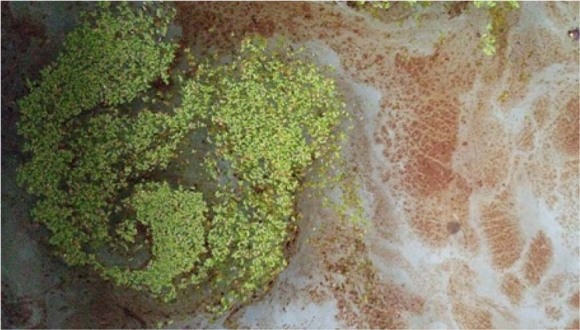
Duckweed mat in oil-contaminated wetlands.

Polyvinyl tanks measuring 0.5 × 0.5 × 0.1 m in length, width and depth purchased from the market were customised into artificial constructed wetlands as a minicosm for the study. Each tank was filled with 7.5 litres of distilled water, reaching 8 cm of the wetlands. From the initial range-finding test, a 10 mL of crude oil (API of 33.51, specific gravity of 0.85) collected with permission from Warri Refinery and Petrochemical Company Limited was drawn using a pipette and introduced into each of the wetlands to simulate an oil spill environment in surface waters (Fig. 2). The replicated setup was allowed to stand for 7 days exposed to the elements. After 7 days, 5 mL of Zyme nutrient solution was added and then 100 grams of acclimated *L. paucicostata* was weighed using a digital analytical weighing balance (Denver Instruments, Model APX-200) and transferred into each of the wetlands except the control (Fig. 3). The setup was monitored for growth and general health conditions of duckweed. Prevailing environmental conditions such as temperature (27 to 34 °C), photoperiod (12 hours) and relative humidity (63 to 67%) were monitored for the study duration.

### Sample collection  and analyses

*In situ* measurements and samples for laboratory analysis were collected bimonthly (every 15 days) for the duration of the study. Total petroleum hydrocarbons (TPH) in crude oil and duckweed tissues were determined using gas chromatography (Shimadzu, Model 6890 N) equipped with a split/splitless injector and a flame ionization detector (FID) following modified standard procedure by the USEPA (EPA 8015 C Revision 2007). Oil samples collected from the treated and control wetlands were extracted using 25 mL of n-hexane from 20 mL of the sample in the wetlands. The extract was agitated for 15 minutes using an ultrasonic device and allowed to settle down for 60 minutes at 28 °C. The filtrate from the extract (about 3 µL) was transferred into vials and was injected into the injector at a temperature of 300 °C using a splitless mode with a relay of about 20 seconds and then analyzed using gas chromatography. The oven temperature was raised from 50 to 300 °C. Inert gas such as helium was used as the carrier gas of the sample through a fused silica capillary column. The GC machine was equipped with an autosampler and flame ionization detector (FID) couple with an HP complete desktop computer system for the analysis. The chromatogram of the analysis was produced using Agilent Chemstation software version 10.

Duckweed samples were collected prior to experimental setup (baseline) and at the end of the study were analyzed for the concentration of hydrocarbons in their tissues. Samples of duckweed were collected from the treated wetlands and oven-dried at 35 °C and allowed to cool down and crushed with a mortar and pestle. Then 100 mg of the crushed sample was taken in a separating funnel and extracted with 25 mL of n-hexane. The above process was repeated to get the maximum yield of hydrocarbons from the sample. The residue from the extract was then dissolved in HPLC grade acetone and the solution was filtered through Whatman filter paper No. 1. The filtrate was filtered through a 0.45 μm syringe. Then 3 μL of the standard and sample solutions were injected into the injector and the chromatogram was recorded.

### Contaminant removal and degradation

Contaminant removal efficiency expressed in percentages was calculated^28^ as shown in Eq. 1;

1 $$R( \% )=\frac{{C}_{o}-{C}_{t}}{{C}_{o}}\times 100$$

where *R* is removal efficiency of contaminant (%), *C*_*o*_ is the initial contaminant level (mg/L), *C*_*t*_ contaminant level at the end of the study (mg/L).

The fraction of TPH bioaccumulated and biodegraded by duckweed was obtained following method described by^2^ in Eq. 2;

2 $$Bio{d}_{C}={I}_{C}-{F}_{C}-{T}_{C}$$

where *Biod*_*C*_ is the concentration of contaminant degraded (mg/kg), *I*_*C*_ is the initial concentration of contaminant (mg/L), *F*_*C*_ is the final concentration of contaminant (mg/L) and *T*_*C*_ is the concentration of contaminant in tissues of duckweed (mg/kg).

### Kinetic rate modelling

The remediation rate of TPH was investigated using the first-order rate kinetic equation^29^ in Eq. (3).

3 $${\rm{In}}{C}_{t}={\rm{In}}{C}_{0}-kt$$

where, *C*_*t*_ is the concentration of parameter at time, t (mg/L), *C*_*o*_ is the initial concentration of parameter at time, t (mg/L), *k* is the first-order rate constant (day^−1^) and *t* is the time (day).

Biodegradation half-life (t_1/2_) is the time needed for a pollutant concentration to degrade to half of the original concentration as seen in Eq. 4^30^.

4 $${{\rm{t}}}_{1/2}={\rm{In}}2/{\rm{k}}=0.6932/{\rm{k}}$$

where, *t*_*1/2*_ is the half-life time, *k* is the biodegradation rate constant.

### Data analysis

Data were analyzed using SPSS version 21 (IBM) and summarized into means, standard errors and percentages, while the level of significance (p < 0.05) was computed using one-way analysis of variance (ANOVA) followed by a posthoc test where significant.

## Results and Discussion

### Growth of duckweed

The growth of *L. paucicostata* in contaminated wetlands shows no obvious inhibition by the concentration of crude oil applied for the study. Although the growth of the plant was slow within the first few days after introduction into the wetlands, duckweed grew to cover almost the entire surface of the setup at the end of the study. No adverse or toxic effect was reported with *L. minuta* for the removal of phenol ranging from 25 to 250 mg/L^31^. Even in studies where macrophytes performance was below expectations^32,33^, the results were unconnected with the inhibition or incapacitation of the species.

### Hydrocarbon removal from wetlands

**Figure 4:**
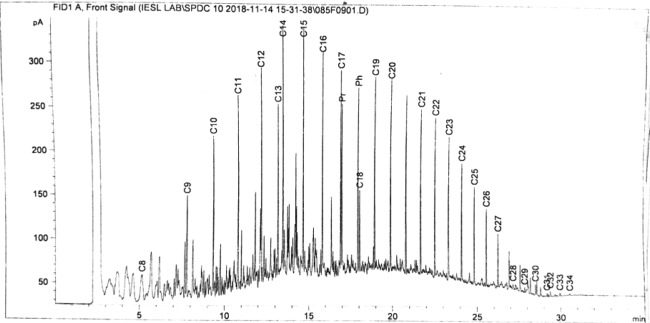
GC profile of TPH in wetland at the beginning of the study.

**Figure 5:**
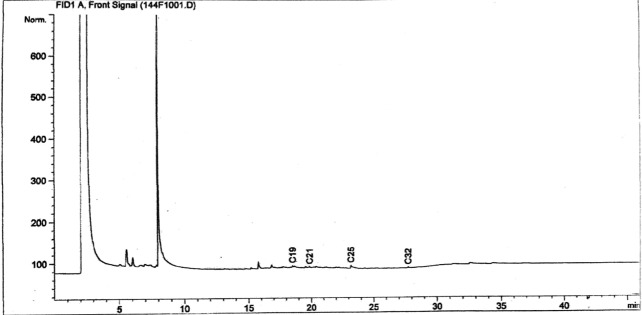
GC profile of TPH in duckweed at the end of the study.

**Figure 6:**
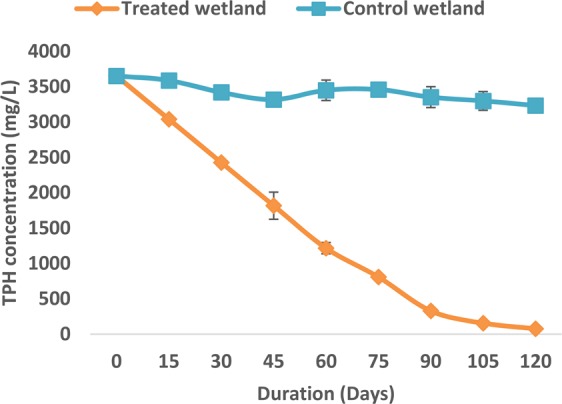
Hydrocarbon removal rate in wetlands with *L. paucicostata*.

The mean concentration of TPH in wetlands contaminated with crude oil decreased significantly (F = 253.405, P < 0.05) by 97.91% (from 3651.77 ± 65.36 to 76.22 ± 6.86 mg/L), while for the control, it decreased by 11.46% (from 3651.77 ± 65.36 to 3233.42 ± 77.07 mg/L) after 120 days of the study (Figs. 4, 5 and 6). Oil on the surface of wetlands provided a maximum and direct contact to *L. paucicostata* for the increased uptake and removal of hydrocarbons from wetlands. Virtually all tissues in duckweed are metabolically active and useful for the potential removal of contaminant from wetlands^17^.

Several studies supported the significant removal of hydrocarbons by various macrophytes including duckweeds from contaminated wetlands^31,34–37^. *L. minuta* removed 100% phenol from wetlands within 21 days (Paisio *et al*., 2017) while *Rhizophora mangle* effected the removal of 87% TPH from contaminated sediments compared to bioremediation with removal of 70% after 90 days^37^. Although microbes present in the rhizome were indicated to enhance removal of hydrocarbons^37^, in other cases, the rhizosphere effects may not enhance the removal of hydrocarbons^35^. A lack of microbial and rhizosphere effect was observed for the transformation products of hexahydro-1,3,5-trinitro-1,3,5triazine (RDX) in hydroponic media^34^. Other studies yielded 72.8% removal of hydrocarbons with *Vetiveria zizaniodes*^38^, 99.9% for phenanthrene with *Scirpus lacustris*^39^, 87.18% and 76.40% for pyrene and benzo[a]pyrene (BaP) with *Acorus calamus*^40^, 94 and 81% for aliphatic and aromatic hydrocarbons with *Azolla filiculoides*^41^ while 81% for chlorobenzotriazole with *L. minor*^9^. Furthermore, *Eichornia crassipes* and *Phragmites* effectively removed hydrocarbons from polluted media^12,42^. Species of macrophytes such as *Alternanthera philoxeroides*, *Panicum hemitomon*, *P. australis* and *Sagittaria lancifolia* successfully remediated South Louisiana crude oil in a wetland setup^43^. Yang *et al*. ^44^ reported that *Pistia stratiotes* and *E. crassipes* were able to remove oil from the media via adsorption and absorption. In a seawater setup, *Laminaria japonica* removed about 90% of phenanthrene and pyrene from contaminated media^36^, while the salt marsh grass *Spartina alterniflora* and *S. patens* increase the biodegradation of spilt oil by transporting oxygen to their roots^6^. The increasing trend in the potentials of selected macrophytes to remove a significant level of organic compounds from contaminated media is vital for the development of pilot wetlands for the treatment of wastewater from industrial activities. Despite the results, studies with water lettuce, *P. stratiotes* and the giant duckweed *Spirodela polyrrhiza* were unsuccessful^23,32^ while black rush performs poorly (15%) in the removal of petroleum hydrocarbons from contaminated sediments^33^. Although macrophytes can remediate pollutants in water, several uncertainties such as plant handling, locality, contaminant behaviour, and other environmental factors could affect the remediation potentials of plants.

Hydrocarbon removal was time-dependent as increased in the duration of the study resulted in increased hydrocarbon removal from wetlands. The removal rate of hydrocarbons from the wetlands was significantly high within the first 60 days of the study and then it gradually reduces towards the end of the study (Fig. 4). This decreasing trend is consistent with previous works as the removal and degradation of TPH and phenol in wetlands is a function of time^31,45^. Within the interval of 15 days that the analysis was conducted, the most pronounced decreased of hydrocarbons was observed between 15 to 30 days (16.78%) of the study and the least was reported between 105 to 120 days (2.15%) respectively. It is safe to indicate that within the first 15 days of the study, the duckweed could be playing a dual role of adjusting to the presence of environmental stress emanating from the hydrocarbon contaminant in the wetland and degrading the hydrocarbon at the same time. The high or rapid uptake and removal of hydrocarbons after the first 15 days is not unusual. The exposure of aquatic plants to organic chemicals results in rapid uptake, sequestration and transformation in plants^36,46^. The significant decrease of TPH in wetlands recorded for the first time with *L. paucicostata* is an indication that this species of duckweed is an efficient phytoremediation agent for the removal of petroleum hydrocarbons from contaminated environment. However, more exhaustive studies are needed to elucidate the removal dynamics of contaminant from wetlands by *L. paucicostata*.

### Hydrocarbon chain reduction

**Figure 7:**
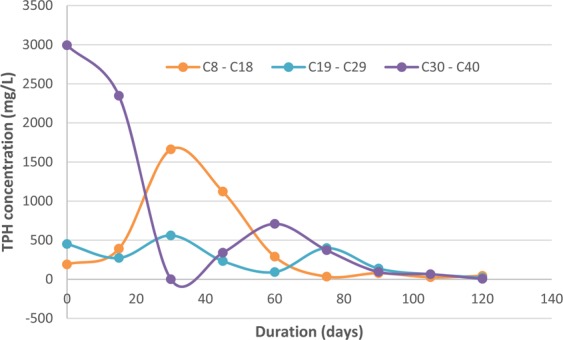
Hydrocarbon chain reduction of crude oil in wetlands with *L. paucicostata*.

Petroleum hydrocarbons consist of a carbon chain backbone ranging from carbon 1 (methane) to carbon 120 (*n*-icosahectane) based on the number of carbon atoms present in the crude oil sample^47^. Lab analysis of the TPH in crude oil showed hydrocarbon chains ranging from C8 to C40 present in the oil sample. This is consistent with hydrocarbons chain present in light crude oil associated with the Niger Delta oil fields^2^. Hydrocarbon chains were categorized into three groups (C8 to C18, C19 to C29 and C30 to C40) for interpretation purposes. The percentage reduction of the hydrocarbon chains in crude oil at the end of the study revealed that C30–C40 (99.84%) had the highest reduction % compared to C19–C29 (95.53%) and C8–C18 (76.61%) respectively (Fig. 7). It is anticipated that duckweed would act first on lower chains hydrocarbons compared to higher chains when exposed to organic compounds, but the reverse could be the case. *L. paucicostata* could preferentially transform higher chains hydrocarbons into lower chains as an adaptive response to mitigate contaminant stress as observed for higher chains hydrocarbons (C30–C40) in this study Moreira *et al*.^37^ reported that *Rhizophora mangle* was able to degrade 82% and 70% of C23–C34 and C24–C40 compared to 63% and 21% of C23–34 and C24–C40 using bioremediation in contaminated sediment.

### Fate of petroleum hydrocarbons in Duckweed

**Figure 8:**
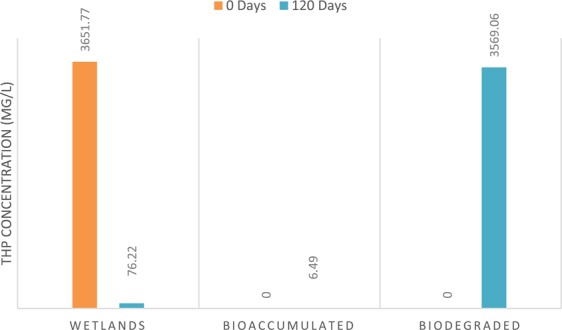
TPH bioaccumulation and biodegradation by *L. paucicostata*.

The transport and fate of hydrocarbons in wetlands was assessed by the estimation of the fraction of hydrocarbons bioaccumulated and biodegraded by *L. paucicostata* (Fig. 6). The concentration of hydrocarbons bioaccumulated (6.49 ± 0.66 mg/kg) by duckweed after 120 days increased significantly (F = 28.115, P < 0.05) by 298.16% compared to the baseline values (1.63 ± 0.64 mg/kg) in plant. The elevated levels of the contaminant obtained in duckweed compared to the baseline further buttressed the point that *L. paucicostata* could be an efficient hyper-accumulator of petroleum hydrocarbons in polluted waters. Assessment of the biodegraded fraction from the initial (3651.77 ± 65.36 mg/L) and final concentration (76.22 ± 6.86 mg/L) of hydrocarbons in wetlands showed that *L. paucicostata* significantly (F = 87.325, P < 0.05) biodegraded 97.74% (3569.06 mg/L) of hydrocarbons in contaminated media (Fig. 8). Any other possible scenario or explanation for the whereabouts of the degraded hydrocarbons fraction other than the sequestration, transformation and possible mineralization in duckweed tissues is difficult to speculate. Previous studies have provided insights on the potentials of aquatic plants including duckweeds to accumulate, sequester and degrade organic compounds^46,48^.

The remediation processes for organic compounds include accumulation, sequestration, degradation, and metabolism of contaminants in plant tissues^48^. During this detoxification process, hydrocarbons are transformed, conjugated, and sequestered^46^. Once the contaminant enters the plant system, it is partitioned to different plant parts^48,49^. High molecular weight organic compounds could be metabolized to secondary and tertiary transformation products rather than complete mineralization in plant^46^. Unlike microbial species that metabolize organic contaminants to carbon dioxide and water, plants use detoxification mechanisms that transform parent chemicals to non-phytotoxic metabolites by enzymes^35,46^. Specific enzymes such as dehalogenase, nitroreductase, peroxidase, oxygenase, laccase and nitrilase have been identified to mediate the degradation and transformation of contaminants in plants^7,48^. These enzymes are capable of transforming organic contaminants by catalyzing chemical reactions^35^ similar to the metabolism of xenobiotics by enzymes in the human liver^46^.

Secondary or tertiary metabolites from crude oil could play a role in plant biochemical processes and may be vital to the growth and development of plants, particularly invasive aquatic plant. *Elodea* sp transformed DDT to DDD, hexachloroethane to perchloroethylene^50^ and atrazine to ammeline^51^. Trichloroethylene taken up by *Populus deltoides* transformed into metabolic components and further degraded to carbon dioxide, chloride ion and water^52,53^, while phenol acted as carbon and energy source for *L. minuta*^31^. Radio-labelled hydrocarbons (methane, ethane, propane, butane, pentane, benzene, toluene, and xylene), benzo[a]pyrene, anthracene and benz[a]anthracene were transformed into polar and non-polar metabolites in plants^35,54,55^.

Despite, a significant uptake and removal of hydrocarbons from the wetland, at the end of the study only 0.2% of hydrocarbon was found in tissues of *L. paucicostata*. Only 0.1% of Bisphenol A (BPA) was found in *Ceratophyllum demersum* despite a significant removal of BPA from local ponds in China^56^. The low accumulation of the contaminant found in the tissues of the *C. demersum* was an indication that BPA was mainly biodegraded by the species^56^. A similar pattern was observed for phenanthrene and pyrene with *Laminaria japonica*^36^ and anthracene, phenanthrene and benzo[a]pyrene with *L. gibba*^24^.

Little is known about the complete transformation and mineralization of hydrocarbons in macrophytes including duckweeds^51^. Uptake of organic compounds depends on the plant species, age of contaminant, and many other physical and chemical characteristics^7,11^. The application of carbon tracer for the identification of intermediate products in the transformation of organic compounds could be the focus of subsequent studies for the remediation of hydrocarbons by duckweed.

### Kinetic rate of hydrocarbon removal in Wetlands

The experimental data were subjected to the first-order kinetic rate equation to model the remediation rate of TPH by duckweed in wetlands (Table 1). The result of TPH remediation considering the rate constant (*k*), half-life (t_1/2_) and the goodness of fit (r^2^) in the treated wetlands and the control could be best fitted into the first-order kinetic rate model. A best line of fit was drawn for the contaminant and r^2^ was found with a coefficient of 0.938 which indicates that the removal of hydrocarbons from wetlands followed reasonably the first-order model. TPH degradation by Ryegrass (*Lolium perenne*) was well fitted with the first-order kinetic model within 90 days^45^. Khellaf and Zerdaoui^57^ indicated that the pseudo-first-order model appropriately followed the removal of contaminants in effluents with *L. gibba*. From the significant coefficient recorded with the first-order model, it is reasonable to infer that *L. paucicostata* is a good candidate for the removal of petroleum hydrocarbons in surface waters.

**Table 1:** First order kinetic rate model for TPH in wetlands.

Parameters	Treatments	1st order model	1st order rate constant (*k*)	t_1/2_ (days)	R^2^
TPH (mg/L)	Treated (wetland)	y = −0.0325(*t*) + 8.6688	0.0325	21.3	0.9379
	Control	x = −0.0008(*t*) + 8.1854	0.0008	866.4	0.7193

## Conclusion

Treating hydrocarbons polluted wetlands using conventional methods attracts concerns from some sections of the society while ecological approaches are considered with limited options presently available to the petrochemical industry. This study has demonstrated that the invasive aquatic plant *L. paucicostata* which is abundant in surface waters across the nation has great potential to ecologically remediate and remove a considerable level of hydrocarbons from crude oil-contaminated waters. The plant has demonstrated its ability to accumulate and degrade hydrocarbons from wetlands. However, due to the complexity of the fate, and transformation/mineralization processes of hydrocarbons by duckweed it was not possible to fully elucidate these processes. It is therefore suggested that studies are required to elucidate the underlying mechanisms of degradation, transformation and mineralization of hydrocarbons by duckweeds.
